# The Evils of Present Methods of Education*Address of the retiring president, before the Western Texas Medical Society, at Its annual meeting in San Antonio, Oct. 26 and 27, 1899.

**Published:** 1899-12

**Authors:** J. S. Lankford

**Affiliations:** San Antonio, Texas


### TEXAS MEDICAL JOURNAL.

ESTABLISHED JULY, 1885.

PUBLISHED MONTHLY.—SUBSCRIPTION $1.00 A YEAR.

VOL. XV. AUSTIN, DECEMBER, 1899. No. 6.

### Original Contributions.

For Texas Medical Journal.

### The Evils of Present Methods of Education.*

BY J. S. LANKFORD, M. D., SAN ANTONIO, TEXAS.

*Gentlemen of the Western Texas Medical Association:*

I have decided to depart from the usual custom, and address you upon a subject not strictly professional, but nevertheless of greater importance, even, than the wonderful progress of medicine and surgery, viz.: Evils of Present Methods of Education. The subject is so broad that I can only hope to touch upon some of the more important points.

Numerous scientific investigators are searching the fertile fields of bacteriology, and the whole world is interested as truth after truth is discovered, but few indeed are the medical men especially who have noticed or tried to check the downward march of our children to physical and mental ruin. The time has come when the thinking physicians of our country must observe the conditions, form conclusions, and ally themselves with the forces that move toward correction of the existing evils, and lend their aid to the establishment of improved methods of education.

First in importance is the necessity of physical development. I

*Address of the retiring president, before the Western Texas Medical Society, at its annual meeting in San Antonio, Oct. 26 and 27, 1899.

maintain that the physical is co-equal in importance with the mental side of life in matters of education. Formerly rural life and the pursuits which led to proper physical development predominated, and our young men and young women grew up strong and healthy. It was unnecessary to look after the physical side of life, because the conditions of living naturally led to the evolution of a perfect man or woman. Free from enervating influences, and exercising abundantly in the open air the child grew up on the farm surrounded by the conditions nature had wisely provided to favor his growth. Everything favored the making of a man.

Now it is not so. The great and growing tendency is to urban life, where nature plays but little part, where the whole scheme of living is contrary to nature's laws. In the city we have not only the weakening influences of vice and dissipation, and the smaller irregularities of living that disturb the vital organs, but the mind is kept in an unceasing stir of interest or excitement leading to morbid development of the whole nervous system. There is a constant and heavy expenditure of nerve energy with insufficient exercise to promote growth of the body and preserve the balance. The history of nations and the lives of individuals have abundantly proven the imperative necessity of equal and symmetrical development of mind and body, and yet we are almost wholly neglecting the physical half of education. There must be a *perfect balance* if we would preserve the health of our children, protect posterity, and maintain our place among nations.

Insufficient and improper diet and irregular eating play a large part in the deficiencies of growth. This, of course, must be looked after in the home. Suitable food, properly cooked, and eaten at regular intervals and with ample time, is very essential, and no school child should be permitted to miss a meal. What, then, can be suggested to improve our schools from the standpoint of physical development?

First, a modern gymnasium is absolutely essential to every school, public and private. The exercises should be compulsory and part of the regular course of instruction. Of course this must be under medical supervision and the exercises wisely adjusted to the requirements of each pupil. The medical supervisor must be a capable man in physical diagnosis, thoroughly know the pupil's condition, carefully restrain some and urge others, and so direct the muscular movements as to develop the weaker parts and promote general health. Much indeed will depend upon the physician.

He should be able to recognize eye strain, and should refer such sufferers to a competent oculist. Patrons do not ordinarily know that a child's life may be ruined by the reflex troubles of unconscious eye strain. This matter is of such urgent importance that it should have attention at once. Every child should be examined at the beginning of school by some one sufficiently qualified to determine whether serious defect of vision exists. It would save much suffering. Seats and desks should be adjustable, and the physician should see that they are properly adjusted to each pupil so the position may be perfect.

He should supervise heating, ventilation, lighting, and insist upon good plumbing, proper sanitary arrangements and the necessary precautions against contagious diseases, especially tuberculosis. Ample space should be provided for each pupil. Our school rooms are crowded unreasonably. Our school buildings haven't sufficient ground. No ordinary school building should have less than from two to five acres, so there may be plenty of room for out-door sport. Old-fashioned play! Give the children plenty of it. They should have at least an hour at noon for luncheon, games, and mental rest. The teachers themselves would be healthier and would get into closer relationship with the pupils if they would join heartily in the games.

It is not a sufficient answer to say, that we can't do these things. We can and must develop the physical side of life, or the evil results will be seen in the next generation, and the same condition of things continuing, will lead us on to ruin. So much for a just balance considered from the physical side.

What shall we say of the death-dealing mental burdens placed upon our children? We impart to the child by heredity and example an ambition to know everything, or, if he doesn't happen to have that ambition we proceed to *hammer* everything into him. We reach out eagerly to grasp and incorporate all of the affairs of the universe in our course of instruction. Regardless of aptitude or natural tendency of mind, we group the pupils in enormous classes and give them all just the same.

Our school boards are composed of able and conscientious men; our superintendents are well qualified and earnest; our teachers are doing harder work than the members of any other profession—more exacting and more irksome than any kind of labor known to me.

The fault is not with any of them. The cause will be found deeper laid than the mere system of any school. It is chiefly: A growing tendency to deeper research. Each branch of learning is expanding so broad and reaching so deep, that it is the study of a lifetime to compass it. And every writer of a text-book is a selfish specialist who wants his pupil to be his disciple and perfect himself in the one branch. And so our children find themselves so hedged about by the requirements of these numerous specialists that they are confronted with the impossible. The fads and fancies of the author are thrown in for further burdens. Then, too, there is much more general information crowded into school life than is necessary.

Gentlemen, the burden is too heavy, and just as sure as effect follows cause in natural law, our generation of children is being weakened and unbalanced, and a still weaker generation will follow. It is but a short step to individual and national degeneracy.

Our great public school system designed to bless the nation will become a national curse. This is not a false alarm or an idle prophecy. Visit the schools and see the careworn teachers always working beyond nature's reasonable limits, and you can better understand why she or he seeks your aid before the middle of the term in order to be able to continue work. And here in this broken-down condition of the teacher we see an index to the heavy course and wrong classification.

One noble young woman in a San Antonio public school teaches 140 little ones—one-half in the morning, the other half in the afternoon. How long can she survive? And what can she do for all these little ones who need the best and greatest lessons at the beginning? Look over the classes of all grades and see the old-looking children; anxiety and age written in their faces, where innocence and youth and joy should shine. Watch the pale, weary little girl trying to make a high mark in eight complex studies. Her chief exercise is carrying home a heavy load of books to study at night. No time to grow! Robbed of all child life. Is it any wonder she is a neurasthenic before she is a woman? And these are to be the mothers of the coming generation! What kind of mothers can they make? What kind of homes? Better for her and better for posterity if she had learned great moral principles and lessons of love at her mother's knee and had never seen a school. Better that she had grown up strong and full of life and hope and the beauty of health, than be a hopeless educated wreck.

The salvation of our boys is that they play more, live more outdoors, shirk their lessons and quit school early to go to work.

We are over-working our teachers; we are crowding the life and hope out of our children. What shall the remedy be? A lighter course, better classification, industrial training, more teachers.

How can the course be reduced? Geography is a fascinating study, but why should our children spend seven years on this branch? The maps are changing every year; widespread intermingling of peoples of diverse habit and custom leads to some change at least in the products of a country; even climate changes.

Let some good authority write a course embracing the essentials which shall cover two years study instead of seven, giving most space to the beautiful study of physical geography. Much of the valuable time spent on this branch might thus be saved. In the same way many other studies could be cut down and simplified.

Some things, indeed, might be entirely eliminated from the course without material loss in education. Give more time to developing the individuality of the pupil and to training him how to think, and less time to stuffing him with a thousand things he will forget before he is grown.

By all means stop night study. School hours are too long already, and any time put in on books at night, especially in children under the fourth grade, is borrowed from the latter end of life.

The facilities for the dissemination of knowledge are rapidly increasing, and our children will become well posted in general information simply by intelligent contact with the world. Leave more to the daily press, greatest if not most accurate of all educators.

Classes should be much smaller and arranged according to mental capacity. The bright pupil can't afford to wait for an idea to reach a dull one, and the dull pupil is embarrassed and hindered by the association. Not only should classes be smaller and arranged to suit the minds of pupils, but the course of study should be suited to the natural fitness of each pupil, so far as it is possible to discern his mental make up.

Industrial training has become an urgent necessity. Competition is so keen that early training is necessary to make a man most successful in any trade, art, or profession. If a boy has not the natural ability to rise above manual labor, why worry him with science, or algebra? Give him the rudiments as far as possible and turn him over to an industrial department, so that he may be trained in the best manner for the avocation which nature intended.

Our girls would be more usefully educated if they had less of books and more training in the arts of cooking, sewing, housekeeping, fancy work—the making and keeping of a home. And these things should be taught in our schools after a sufficient number of books have been dropped from the course.

The system of marking in most schools takes up the teacher's time, and cannot be otherwise than harmful to the pupil. The strain to make high marks is terrific, and keeps the child in a state of anxiety all the time, much to the damage of the nervous system, and this practice should be abolished. Grades should be arranged not according to examination marks so much as on the judgment of able and conscientious teachers.

Finally, our teachers are struggling under impossible burdens. We need more teachers, teachers fitted by nature with great qualities, and paid according to the greatness of their calling. Free them from the slavery of an incessant effort to pass to a higher grade. Once a teacher, always a teacher—they naturally develop with experience, and should be graded more with reference to general fitness than text-book knowledge. Then, with smaller classes and less burdensome tasks they would have opportunity to study the natural bent and the needs of each pupil to a much greater advantage. The teacher who can rise above all set methods and impart the best and greatest lessons, physical, intellectual, moral, and spiritual, uninfluenced by prejudice, is the greatest benefactor of this age, not excepting any.

In conclusion, gentlemen, I appeal to you as guardians of the public health, interested in the future of our people and our country, to give this subject some thought, and to use your influence for proper reform.

When our schools are so arranged that the body, the noble temple of the spirit, shall have co-equal education with the mind; when the child is symmetrically developed in mind, body and soul, and the best that nature has given him is utilized, then, indeed, may we be assured that our race will be strong, that our people will be healthy and prosperous, and that our nation will be perpetuated.

